# The Unfulfilled Potential of Synthetic and Biological Hydrogel Membranes in the Treatment of Abdominal Hernias

**DOI:** 10.3390/gels10120754

**Published:** 2024-11-21

**Authors:** Kenigen Manikion, Christodoulos Chrysanthou, Constantinos Voniatis

**Affiliations:** 1 Laboratory of Nanochemistry, Department of Biophysics and Radiation Biology, Semmelweis University, Tűzoltó Street 37-47, H-1094 Budapest, Hungary; 2 Department of Surgery, Transplantation and Gastroenterology, Semmelweis University, Üllői Street 78, H-1082 Budapest, Hungary

**Keywords:** surgical meshes, bilayer membranes, mechanical studies, in vivo studies, tissue engineering

## Abstract

Hydrogel membranes can offer a cutting-edge solution for abdominal hernia treatment. By combining favorable mechanical parameters, tissue integration, and the potential for targeted drug delivery, hydrogels are a promising alternative therapeutic option. The current review examines the application of hydrogel materials composed of synthetic and biological polymers, such as polyethylene glycol (PEG), polyvinyl alcohol (PVA), gelatine, and silk fibroin, in the context of hernia repair. Overall, this review highlights the current issues and prospects of hydrogel membranes as viable alternatives to the conventional hernia meshes. The emphasis is placed on the applicability of these hydrogels as components of bilayer systems and standalone materials. According to our research, hydrogel membranes exhibit several advantageous features relevant to hernia repair, such as a controlled inflammatory reaction, tissue integration, anti-adhesive-, and even thermoresponsive properties. Nevertheless, despite significant advancements in material science, the potential of hydrogel membranes seems neglected. Bilayer constructs have not transitioned to clinical trials, whereas standalone membranes seem unreliable due to the lack of comprehensive mechanical characterization and long-term in vivo experiments.

## 1. Introduction

**Figure 1:**
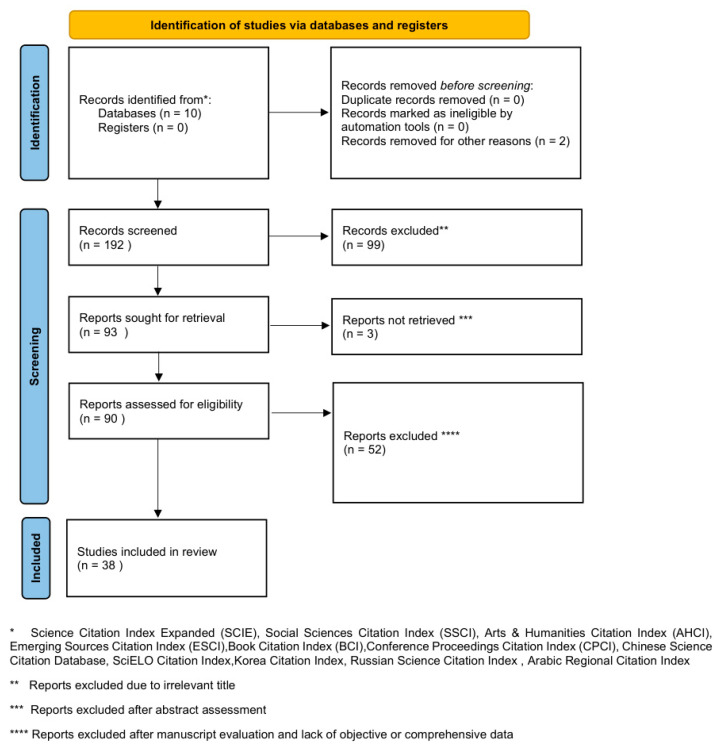
The current literature review PRISMA 2020 flowchart [3].

Hydrogel membranes are a unique, yet often overlooked material. Hydrogels have several advantages, including a three-dimensional self-sustained structure, a high water content, tissue-like elasticity, and permeability, which allows for small molecules to diffuse freely through them [1,2]. These features make them especially attractive for researchers aiming to fabricate scaffolds for tissue regeneration. Additionally, their physical, chemical, and mechanical properties can be optimized for cell adhesion, proliferation, and differentiation. Even more so, they can also be functionalized and designed to carry drugs or active ingredients, which can enhance the specific application in mind [1,2]. Interestingly, the utilization of hydrogel membranes for the treatment of abdominal hernias is not a novel notion. The field, however, seems to have reached an invisible obstacle. Why can hydrogel membranes not take over the conventional materials? What are we missing, and how can we take the next step toward the industrialization of theses membranes? In the following review, we aim to shed some light on the current difficulties and prospects of hydrogel membranes by answering these intriguing questions. The reporting of this review was performed according to the standards of the Preferred Reporting Items for Systematic Review and Meta-Analysis (PRISMA), as seen below (Figure 1).

## 2. A Brief Overview of Abdominal Hernias

**Figure 2:**
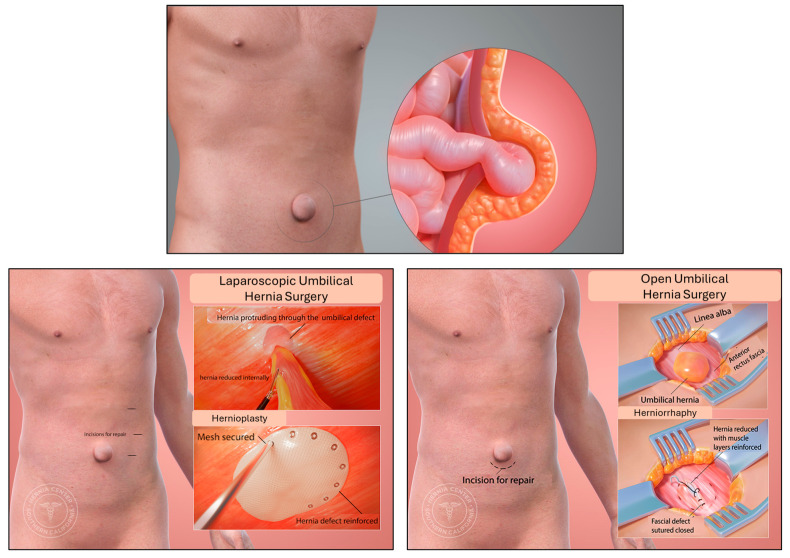
An umbilical hernia and its treatment options. (Used with permission from the Hernia Center of Southern Carolina, USA).

An abdominal hernia is defined as the pathological herniation or protrusion of abdominal visceral tissues through a defect on the abdominal wall. Being a purely mechanical issue, hernias develop due to an imbalance between intra-abdominal pressure and the strength or integrity of the abdominal wall [4]. The risk factors for hernia formation include heavy weightlifting, age (>65 years), obesity, atherosclerosis, smoking or pulmonary disease, tumors, pregnancy, and even genetic disorders affecting connective tissues such as Ehlers–Danlos syndrome [4]. A hernia is perhaps the most common medical condition requiring surgery worldwide, affecting millions of people, with an incidence rate of almost 30% (27% of males; 2% of females) [5]. Hernias can be classified according to location (umbilical, inguinal, femoral, etc.), etiology (congenital or acquired), their contents (adipose tissue, the intestinal loop, the appendix, etc.), and their complexity (simple-reducible, recurrent, incarcerated, or strangulated). After decades of medical research, it has been repeatedly proven that the only definitive treatment is surgery [4]. The surgical repair of abdominal hernias has gone through several stages of evolution in terms of both the surgical techniques and the utilized instruments and devices [6]. Currently, the standard operation of care involves the low-tension closure of the abdominal wall with a surgical mesh [7,8]. Simply put, the mesh closes the defect and prevent recurrence. The surgical operation can be conventional, i.e., open surgery, laparoscopic, minimally invasive (single port), or robot-assisted [7,8,9] (Figure 2). Complications do not only occur due to iatrogenic mistakes (bleeding, chronic pain, infection, etc.), but also due to an extreme inflammatory reaction to the surgical mesh causing visceral (bowel) adhesion. Such complications include chronic pain, mesh folding, mesh migration, infection, and even intestinal perforation [10,11,12,13].

## 3. A Brief Overview of Surgical Meshes

At first, hernias were treated without meshes (tension repair). Although initially surgeons were divided regarding their usage, decades of research has proven that the benefits of surgical meshes outweigh their drawbacks [14]. The currently used surgical meshes have two functions. First, to preliminary provide the abdominal wall with mechanical support and relieve any tension which can lead to pain, bleeding, and necrosis. Second, to induce an inflammatory reaction, which will lead to granulation tissue formation, collagen deposition, and new connective tissue development [6] that will prevent future hernia recurrence. It is important to clarify that the abdominal wall will only regain about 75% of its original strength [15]. This phenomenon is more pronounced in the case of larger hernias. It should be noted that as the body heals, it is primarily the newly formed connective tissue that will provide mechanical support; and not the mesh itself. This is the reason most of these meshes are macroporous [6,15,16]. By allowing the inflammatory reaction to occur and granulation tissue to pass through, the mesh can be somewhat integrated into the abdominal wall. Probably the weakest component of this arrangement is the strength of mesh–abdominal wall adhesion. The sutures, clips, or fibrin glue that are typically used to fix the mesh do not provide satisfactory mechanical support by themselves [17]. Evidently, as the main concern is hernia recurrence, the currently used surgical meshes are primarily focused on the mechanical performance and the inflammatory reaction.

**Figure 3:**
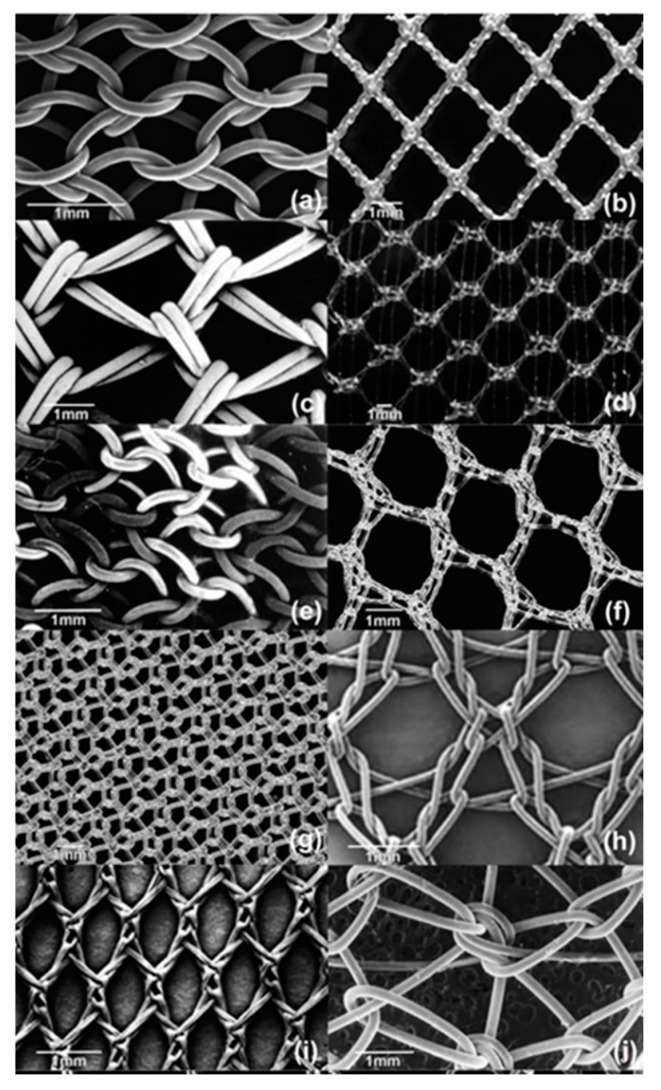
Examples of synthetic meshes. (**a**) Bard Mesh, (**b**) Vypro, (**c**) Prolene V R, (**d**) Bard V R Soft Mesh, (**e**) Trelex V, (**f**) Optilene V R, (**g**) SurgiPro V R, (**h**) Parietene V R, (**i**) Mersilene, and (**j**) Dynamesh V R IPOM. Used with kind permission from Elsevier [20].

Several types of meshes exist (Figure 3). These are primarily categorized by the material’s origin, i.e., synthetic, biological, composite, or hybrid [6,15,16]. In addition, they are also frequently grouped according to density, porosity, and biodegradability (Table 1).

### 3.1. Synthetic Meshes

Synthetic meshes are unequivocally dominating the field. These meshes are typically produced via the melt extrusion of high-molecular-weight thermoplastic polymers. After extrusion, additional processing is frequently performed (e.g., weaving and braiding), resulting in extremely strong, yet dry and rather rigid macro- or microporous meshes (Figure 3). The main options include the following:

*Polypropylene Meshes:* Undoubtedly the most commonly used meshes due to their unparalleled mechanical properties. Their extreme inflammatory reaction provides effective long-term reinforcement to the abdominal wall. However, they have been associated with severe complications, including chronic pain, adhesion formation, mesh migration, and intestinal perforation [12,13].

*Polyester Meshes:* Similar to polypropylene, polyester meshes have excellent tensile strength. Polyester has not been as popular as polypropylene. Interestingly, it is the first choice in some European countries (e.g., Spain and Portugal). Upon fixation, polyester meshes induce rapid fibroblastic infiltration, resulting in less mesh shrinkage. For this exact reason, if placed intra-abdominally, they are unfortunately more prone to visceral (bowel) adhesion. Some reports also describe unregulated degradation and a loss of strength over time, along with higher infection rates [17,18,19,20,21].

*Polytetrafluoroethylene Meshes:* According to some studies, these meshes result in less inflammation and adhesion formation compared to polypropylene or polyester meshes. Consequently, the reduced inflammatory reaction results in poor tissue integration and higher hernia recurrence rates. Furthermore, several studies have documented that polytetrafluoroethylene is more susceptible to infection than other materials [15,18,19,20,21].

*Polyvinylidene Fluoride Meshes:* These meshes are composed of a non-absorbable fluoropolymer and exhibit similar tensile strength and surface characteristics to polyester. In contrast, they are more resistant to hydrolysis, degradation, and stiffening. Unfortunately, the lack of clinical trials and long-term data do not support the use of PVDF instead of other meshes [15,18,19,20,21].

### 3.2. Biological Meshes

**Figure 4:**
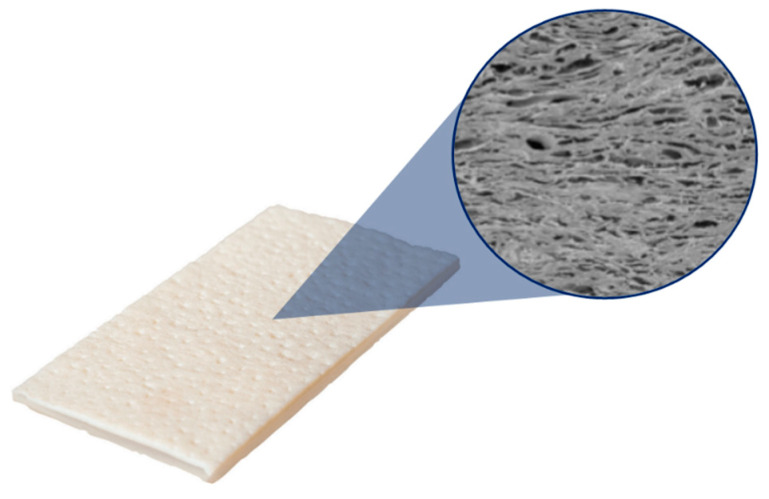
An example of a biological mesh: an Acellular Dermal Matrix (Allomend by Allosource, https://hcp.alloderm.com/, accessed on 10 November 2024).

While synthetic meshes are dominating the market, their non-degradable nature, extreme inflammatory reaction, and post-operative complications have raised numerous concerns [11]. For this very reason, some researchers started to explore biologically derived materials. Biological meshes can be developed from human (allograft) or animal (xenograft) tissues. Most of them are produced by the decellularization of various tissues, resulting in an acellular complex collagen matrix. The sources include human dermis, porcine dermis, intestine and bovine dermises, or the pericardium [16] (Figure 4). The meshes are also produced from artificially synthesized biological polymers, for example polyglactin or polylactic acid. These are typically found in composite meshes (see next section).

Due to their biological origin, they are known to cause allergic reactions. Biological materials induce foreign body reactions to a lesser degree than synthetic ones; thus, the risk of complications due to visceral adhesion formation is also decreased [18]. However, due to their poor mechanical properties, the hernia recurrence incidence is higher. To combat their poor mechanical performance, researchers have explored cross-linking strategies. Although cross-linking indeed enhances the mechanical properties of meshes, it concurrently slows down, or even completely prevents biodegradation [22,23,24]. Therefore, the efforts to make them more durable often take away their innate biocompatibility/biodegradability advantages [25]. Furthermore, cross-linking has been associated with increased toxicity and inflammation due to the presence of an unbound cross-linking agent [2,22,23,24]. Unfortunately, due to their more complicated and expensive fabrication, only a small number of clinical studies have been carried out; therefore, no significant evidence exists to support their usage. Overall, their poor mechanical performance, higher recurrence rates, and excessive costs make biological meshes a secondary option to synthetic meshes.

### 3.3. Composite and Hybrid Meshes

These meshes are made from bioabsorbable or biodegradable, synthetic, and/or biological materials. The concept behind their fabrication is to provide temporary support until the body’s own tissues can take over. As the aim is to optimize the inflammatory reaction and limit visceral adhesion formation, their long-term durability is limited, and the risk of hernia recurrence is higher compared to that of their purely non-biodegradable “competitors” [18,26,27]. In addition, they are very expensive compared to the cost of the standard polypropylene choices.

Absorbable (bioabsorbable) meshes are typically made from polyglycolic acid and/or polyglactin 910 (polyglycolic acid copolymerized with lactic acid). These meshes are absorbed within 90–180 days and lose 50% of their original tensile strength within 2–10 weeks post-implantation. Due to the higher hernia recurrence incidence, they are not favored amongst surgeons [18,26,27].

The biodegradable meshes include a number of options: Bio-A is composed of trimethylene carbonate and polyglycolic acid and can maintain 70% of its original tensile strength for 3 weeks. Safil is made of modified polyglycolic acid and can maintain 50% of its tensile strength for 20 days, while full absorption occurs between 60 and 90 days. The TIGR Matrix is a very popular, upcoming mesh made of polyglycolic acid and polylactic acid microfibers. Its advantage compared other biodegradable meshes, is its tensile strength that provides stable mechanical support for at least 6 months after surgery. Biodegradation starts at 9 months (degradation of the polyglycolic acid fibers), while full absorption occurs at approximately after two years. Phasix is another example of a bioabsorbable mesh that is fabricated from poly-4-hydroxybutyrate. Its complete absorption occurs at 52 weeks. A composite version also exists with a carboxymethylcellulose and hyaluronic acid coating [18,28] for better wound healing and enhanced tissue integration. Overall, theses meshes are deemed safer, resulting in less hernia recurrence compared to biological meshes. However, due to their high cost combined with the low number of clinical trials supporting their usage, they are only used for special surgical indications.

### 3.4. Issues and Challenges with Current Hernia Meshes

The search for an ideal hernia mesh seems unending. The current hernia meshes, particularly those made from polypropylene—while dominating the clinical field—could be considered as a double-edged knife. From a mechanical point of view, these meshes are outstanding, completely overachieving the required minimal mechanical strength. Even more so, some studies have questioned whether these meshes need to be as strong. In the long term, it is not mesh’s tensile strength that maintains the integrity of the wall, but the strength of the newly formed connective tissue. Inducing controlled tissue regeneration, however, is not a simple task.

Although generated inflammation has its purpose (new connective tissue formation), due to foreign body reactions, visceral adhesion can occur, which then can endanger the surrounding anatomical structures (an intestinal obstruction, an abscess, peritonitis, etc.) [6,11,28,29]. Furthermore, the encapsulation of the mesh in a fibrous capsule, which then can calcify, is also a potential complication as seen for the example in case of breast implants [30]. On the contrary, biological meshes are indeed biodegradable and integrate better with the local tissue, but their poor mechanical performance and extreme inflammatory reaction remain a major concern.

**Figure 5:**
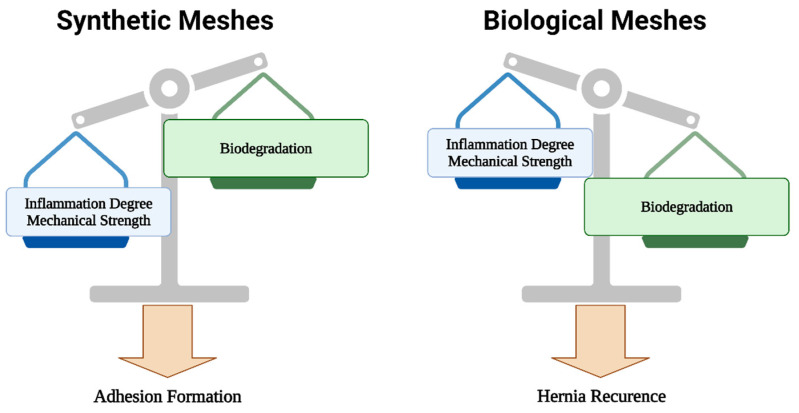
Advantages and disadvantages of synthetic and biological meshes.

Therefore, on the one hand, we have strong synthetic meshes that induce excessive inflammation and are prone to adhesion formation and other complications, while on the other hand, we have biological meshes offering better biocompatibility and biodegradability, yet higher hernia recurrence rates (Figure 5). Finally, composite biodegradable meshes are quite expensive, resulting in a scarcity of long-term, large population clinical studies that would potentially promote their everyday usage and justify their excessive costs.

## 4. Hydrogel Membrane Utilization in Hernia Treatment

Hydrogels are three-dimensional, cross-linked polymer networks that can absorb and retain substantial amounts of the surrounding water or biological fluid, while maintaining their structural integrity. Due to their high water content, soft consistency, and elastic nature, they can be designed to very accurately resemble human tissues [1,31,32]. Possessing several advantageous features, hydrogel membranes have been extensively investigated as drug carrier systems and tissue engineering scaffolds. They are also described as highly versatile, as the physical and chemical properties can be tailored and fine-tuned to the specific requirements of the tissue they intend to replace. Interestingly, hydrogels materials are not commonly known or utilized by clinicians. In addition, they are often overlooked by the surgical community due to their poor mechanical performance. This misconception hinders any significant progression in the field, as evidenced by the number of clinically relevant manuscripts published in recent years. Unknown to many, hydrogels truly possess potential which has yet to be exploited. In terms of hernia treatment, four main advantages can be identified:

First, their innate flexible nature can be a serious advantage. When compared to the conventional meshes, surgical manipulation and hydrogel membrane positioning during laparoscopic surgery is simple and fast. Laparoscopic surgical mesh manipulation and fixation has been a debate topic for years. Different approaches can be found in the relevant literature as to how one can perform this crucial surgical step [17]. The importance of this step is also evident by recent innovations such as magnetic and balloon-assisted hernia meshes [33,34]. In contrast to the rigid and difficult-to-handle conventional meshes, a flexible self-supported hydrogel membrane could simplify placement and decrease the operative time [35].

Second, their soft nature could result in fewer post-operative complications. Mesh folding, migration, and even tissue perforation are some mesh complications which could be avoided by using a soft material instead of rigid meshes. Post-operative complications could also be minimized by the proper chemical modification of the applied polymer, reducing visceral adhesions [2,36,37,38].

Third, the structure and nature of hydrogel membranes offers an unparalleled option for tissue regeneration. Compared to the conventional meshes, biodegradable hydrogel membranes not only provide support, but also a better template for tissue regeneration. Due to their innate hydrophilic nature, protein adsorption, and therefore immune cell activation, is also decreased. This, in turn, can also lower the degree of giant cell production, inhibiting the foreign body reaction, and hindering implant encapsulation. Furthermore, hydrogels can be fabricated with materials inducing cell attachment (e.g., polyglactin), or can even be combined with cell adhesion ligands (e.g., RGD peptides) [36,37,38]. When this feature is combined with tissue-like elasticity, a high water content, and favorable porosity, hydrogels become penetrable scaffolds where the cells can reach the core of the implant, making 3D colonies. If the material is fully biodegradable, the entire hydrogel can be consumed by local tissue, achieving true tissue integration. Interestingly, based on the rigidity and water absorption degree of the scaffold, even cell differentiation can be modulated, resulting in differentiation from the fibroblasts to the chondroblasts, etc. [36,37].

Finally, perhaps the biggest advantage of hydrogel membranes is their drug carrier potential. From simple antibacterial agents to analgesics or tissue growth promoters, the options are limitless [39,40]. In this era of medicine, optimizing recovery and decreasing the hospitalization duration are in the focus of surgical care. A large number of hernia surgeries are currently performed in the so-called one-day surgery setting. In other words, the patient is admitted; surgery is performed in the morning; and in the afternoon, the patient is discharged and can go home. A smart material with antibiotic or analgesic drug-release capabilities would result in safer implants, further decreasing the need of hospitalization.

## 5. Hydrogel Membrane Fabrication and Cross-Linking

**Figure 6:**
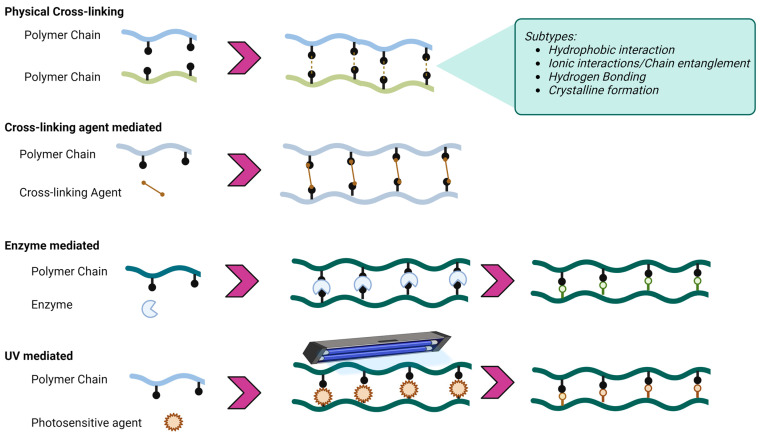
Examples of cross-linking tactics [45,46].

Hydrogel fabrication is not always a simple matter. It can be a straightforward process, such as preparing a polymer solution, which is then freeze-thawed. It can also involve more complicated steps, such as enzyme addition, UV light usage [2,41,42], or even the utilization of the popular electrospinning technique [42]. Ultimately, cross-linking determines the mechanical, chemical, and biological properties of membranes [43]. Furthermore, the type of cross-link, i.e., physical or chemical, and the cross-linking degree will regulate the absorption of surrounding liquid and the swelling degree, the porosity, and the degradation time [44]. The specifics of this topic are not the focus of this manuscript, and literature reviews on this topic. In addition, reviews on this topic have been published before, most recently by Denisa Radulescu and Weikang Hu et al. [44,45]. As a brief overview, the main cross-linking strategies can be found in the following table and schematic figure (Table 2, Figure 6).

Although the cross-linking strategy is important, the choice of polymer is paramount. Several polymers have been investigated as hernia treatment materials. Synthetic or biological, each polymer has distinct advantages and disadvantages. The most popular options include the following:

**Polyethylene Glycol (PEG)**: PEG is a synthetic, hydrophilic, and biocompatible polymer widely used in medical hydrogels due to its excellent water solubility and non-toxicity. PEG provides a stable platform for drug delivery and tissue engineering. However, PEG hydrogels require cross-linking to improve their mechanical properties, and thus may not be optimal for load-bearing applications [40,47].

**Polyvinyl Alcohol (PVA)**: PVA is a synthetic polymer known for its biocompatibility and non-toxicity. PVA-based hydrogels are often used as components of drug capsules in tissue regeneration and wound-healing applications. Cross-linking by a physical or chemical method is necessary to fabricate insoluble and mechanically strong hydrogels. PVA hydrogels are known for their anti-adhesion properties [32,48].

**Polysaccharides**: This category includes cellulose, chitosan, and hyaluronic acid. These natural polymers are able to absorb water and form a hydrogel post-implantation. They are known for their biocompatibility, biodegradability, and ability to mimic the extracellular matrix (ECM). In addition to these characteristics, they can also exhibit some extra features which render them ideal for several medical purposes. Chitosan, for example, provides antimicrobial properties and is widely used in wound healing and soft tissue regeneration, while hyaluronic acid promotes tissue hydration and cell proliferation. These polymers are excellent candidates for soft tissue repair [49,50].

**Polyacrylamide (PAAm)**: PAAm is a synthetic polymer that forms stable hydrogels. While it has excellent mechanical properties, its biocompatibility is a concern. In biomedical applications, PAAm is often copolymerized with other monomers to reduce toxicity and enhance biocompatibility, making it a viable candidate for hydrogel membrane fabrication [51].

**Poly(N-isopropyl-acrylamide) (PNIPAM)**: PNIPAM is a temperature-sensitive polymer that forms hydrogels at body temperature. Its thermoresponsive nature allows it to be used as an injectable hydrogel, making it particularly useful in minimally invasive surgeries and drug delivery systems [52].

**Poly (lactic-co-glycolic acid) (PLGA)**: PLGA is a biodegradable and biocompatible polymer frequently used in tissue engineering and drug delivery. Its degradation rate can be tailored to meet specific needs, which makes it an ideal choice for scaffolds that require precise control over the timing of degradation and tissue replacement [53,54].

**Gelatin**: Gelatin is a biopolymer derived from the hydrolysis of collagen. It is typically sourced from bovine or porcine tissues, such as bones and skin. It is used in hydrogel membrane fabrication due to its biocompatibility, biodegradability, and ability to promote cell adhesion and proliferation. Gelatin hydrogels are widely applied in soft tissue engineering, but they require cross-linking to improve their mechanical stability and prevent rapid biodegradation [31,55].

**Silk Fibroin**: Derived from Bombyx mori silkworms, silk fibroin is a biocompatible and biodegradable protein that provides excellent mechanical strength, making it ideal for applications, such as skin, bone, and cartilage regeneration. Silk fibroin scaffolds closely resemble the natural extracellular matrix (ECM), thus promoting cell proliferation and differentiation. Silk fibroin hydrogel have been investigated for wound healing, drug delivery, and tissue scaffolding applications [56,57].

**Extracellular Matrix (ECM)**: Derived mainly from animals, this material provides an effective scaffold for tissue regeneration by resembling the natural ECM found in human tissues. This material has been utilized for wound healing and soft tissue repair due to its ability to support cell adhesion, proliferation, and differentiation. It can induce an allergic reaction; therefore, it requires purification to minimize the risk of immune rejection [58,59].

Each of these options offer unique properties that can be advantageous for hernia repair applications. However, challenges such as poor mechanical strength, biodegradability, and biocompatibility are common. These limitations can often be addressed by combining polymers or applying chemical modifications to optimize the performance.

## 6. Advances in Hydrogel Membrane Utilization

Hydrogel utilization can be summarized into two main strategies. The first is to utilize hydrogels as a complementary component, covering the primary material (typically a synthetic surgical mesh), forming a bilayer system. The second is to fabricate a standalone hydrogel membrane with all the advantageous properties of a hydrogel that hopefully can be strong enough to be considered for hernia treatment.

### 6.1. Bilayer Constructs

The aim of a bilayer system is either to decrease the number of common post-operative complications, such as visceral adhesion, or add advantageous features, such as tissue integration and antibacterial effects. A common benchmark for these studies is to perform mechanical and biological characterization. As seen in the table below (Table 3), these studies typically carry out mechanical tests to determine tensile strength by uniaxial pulling or, include compression and cyclic tests to evaluate durability and strength. From a biological aspect, in vivo studies are performed to evaluate biocompatibility, predominantly using rodent models (e.g., Wistar rats or Sprague-Dawley rats). A standardized protocol for surgical mesh biocompatibility and applicability, including the implantation site and study duration, has not been established. Due to the exact same reason, long-term studies exceeding the 6-month time interval are limited, just as large animal experiments are scarce.

Overall, bilayer systems perform better than the conventional meshes. The lack of mechanical strength is not an issue, as the synthetic component provides more than enough support. Furthermore, many of the aforementioned systems emphasize improved biocompatibility and significantly reduced inflammatory responses (Table 4). Furthermore, the addition of hydrogels has also been correlated with decreased adhesion formation and a lower incidence of post-operative complications.

**Figure 7:**
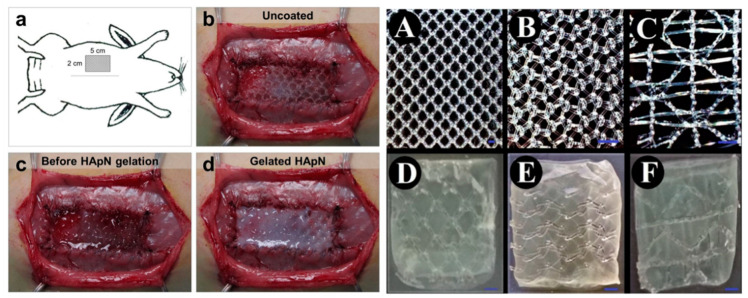
Examples of bilayer constructs utilizing poly(N-isopropylacrylamide) hyaluronan derivative (**left**, (**a**–**d**) [63] and cellulose (**right**, (**A**–**C**) uncoated meshes/(**D**–**F**) bacterial cellulose-coated meshes) [84].

Several meshes (Figure 7), including those incorporating Sirolimus (SRL) hydrogel, alginate, and bacterial cellulose with chitosan are specifically designed to reduce adhesion formation, a critical factor in post-surgical recovery [29,64,78,80]. However, some combinations like pectin–honey hydrogels showed no significant reduction in visceral adhesion formation [22,57]. Bilayers integrated with Aldehyde Bletilla striata polysaccharide (BSPA)-modified chitosan and ECM from pig skin are designed to promote wound healing and tissue regeneration, facilitating better integration with the surrounding tissues and improving the overall healing outcomes [63,66,74,77]. Finally, some meshes, particularly those with methacrylinated gelatin, nanosilver, or porcine gelatin with methacrylic anhydride (GelMA), offer additional benefits, such as antimicrobial activity and antioxidant properties, which help prevent infections and ensure the longevity of implants [75,79,82].

**Figure 8:**
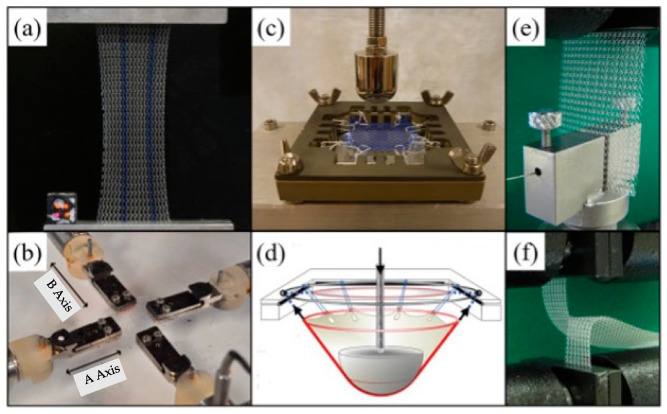
Evaluation methods for mechanical biocompatibility of surgical meshes: (**a**) uniaxial tensile, (**b**) biaxial tensile test, (**c**,**d**) ball bursting and deformation of mesh in ball burst testing, (**e**) suture retention, and (**f**) tear test [83].

On the other hand, we believe that it is important to address the fact that uniaxial mechanical testing does not accurately replicate the mechanical load which the construct will be subjected to after implantation. More comprehensive examination methods, such as biaxial tension, ball burst, suture retention, and tear resistance [83], are not commonly reported. This is a critical issue as laboratory and clinical settings are far from similar. Fortunately, several options are available (Figure 8) that can be utilized to assess the meshes in settings more relevant to clinical application. Furthermore, studies with extended in vivo durations (over 6 months) are very limited. This seems rather oxymoronic since biocompatibility and tissue integration should be the primary focus. In addition, large animal model studies are also limited.

Apart from polypropylene, other synthetic and non-biodegradable systems have been investigated. Polyester meshes have been combined with PVA, γPGA, and gelatin hydrogels to improve flexibility and biocompatibility [37,85] (Table 5). The PTFE meshes have been combined with bovine bone-derived gelatin. These meshes were tested in mice, focusing on subcutaneous implantation and its effects on tissue integration. Finally, polyurethane electrospun meshes have also been investigated in combination with methacrylinated gel, showing promising results [82].

The polyester bilayers showed a significant boost in mechanical strength and were durable enough to withstand sutures and surgical manipulation. Moreover, these meshes provide additional benefits, such as inhibiting targeted microorganisms, promoting the expression of collagen type I, and accelerating wound closure by facilitating fibroblast cell migration. On the other hand, their tensile strength is less than that of their polypropylene counterparts. PTFE meshes are particularly notable for their ability to enhance angiogenesis. This increased formation of blood vessels is crucial for ensuring proper tissue integration and healing, making these meshes well suited for applications requiring robust vascularization and tissue support. Polyurethane meshes also benefit from a methacrylinated gel cover (decreased adhesion formation); however, no data have been documented regarding their mechanical performance. Overall, the same issues are present as previously seen with polypropylene constructs, namely the lack of comprehensive mechanical characterization and short duration in vivo animal experiments (Table 6).

### 6.2. Standalone Hydrogel Membranes

The second option for hydrogel utilization is standalone hydrogel membranes. Although limited, these studies prove that hydrogels do not necessarily need to be combined with non-degradable thermoplastic polymers that take away from their advantageous nature. These hydrogels can be divided into synthetic and biological hydrogel membranes, as seen in Table 7 and Table 8, respectively.

Synthetic hydrogels include poly(vinyl alcohol) membranes, very popular polymers known for being bioinert, but also exhibiting excellent anti-adhesive properties and N-isopropylacrylamide-N-vinylpyrrolidone, a very unique material which is designed to be injectable and also thermoresponsive [88].

**Table 7:** Experimental information of selected synthetic standalone hydrogel membranes (N/A: not available or not performed).

Polymer	Cross-Linking Agent	Mechanical Studies	In Vivo Studies		Ref.
			Model	Duration	
Poly(vinyl alcohol)	Glutaraldehyde	N/A	Wistar Rats Abdominal wall incision Domestic Pigs Intra-abdominal position	90 days: 5 weeks	[35]
		Uniaxial tester (≈0.3 Nm^2^/g)	N/A	N/A	[42]
Poly (NIPAAM-co-VP-co-MAPLA)	N/A	ElectroForce 3200 Series II (Bose) (≈275 KPa)	Lewis Rats Injections	21 days	[89]
Poly(vinyl alcohol)	HCl	Uniaxial tester (17 N)	Wistar Rats: Abdominal wall incision	6 months	[90]
	Dimethyl sulfoxide	Tensile Test (1.3 MPa)	Wister Rats: Abdominal wall incision	28 days	[91]
	Freeze thawing	Tensile Test (2.5 MPa)	Wistar Rats: Abdominal wall incision	45 days	[88]

**Table 8:** Experimental information of selected biological standalone hydrogel membranes. (N/A: not available or not performed.)

Polymer	Cross-Linking Agent	Mechanical Studies	In Vivo Studies		Ref.
			Model	Duration	
Gelatin and Acid Anhydride (GelMA)	UV irradiation	Stretch Stress (≈30 Kpa)	Rabbits Abdominal wall incision	8 weeks	[39]
Gelatin and Poly (g-glutamic acid)	Genipin	Uniaxial tester (≈11 MPa)	Sprague-Dawley Rats: Dorsal implantation	12 days	[85]
Decellularized ECM		Rheometer Discovery HR 10 (≈100 Pa)	Immunodeficient Mice Median Superior incision	7 days	[92]
Acellular dermis grafts and Tyramine-substituted hyaluronan (THA)	H_2_O_2_	N/A	Wister Rat intraperitoneal implantation	1–4 weeks	[93]
Hyaluronic acid (HA) derivative	Oxime bonding from alkoxyamine-terminated Pluronic F127	HAAKE MARS III rheometer (3000 Pa)	N/A	N/A	[41]
	Calcium Alginate	CVO 120 stress controlled Rotational Rheometer	Wistar Rats Abdominal wall incision	14 days	[94]
Resilin	N/A	Tensile Test (≈550 Mpa)	Rats: Abdominal wall incision	6 weeks	[95]
Silk fibroin (from Raw *B.mori*)	N/A	Tensile Test (≈5.96 Mpa)	Sprague-Dawley Rats: Ventral implantation	28 days	[96]
Bovine collagen and (poly (lactic co-glycolic acid)	N/A	N/A	Nu/nu Rats: Dorsal Implantation	21 days	[97]

**Figure 9:**
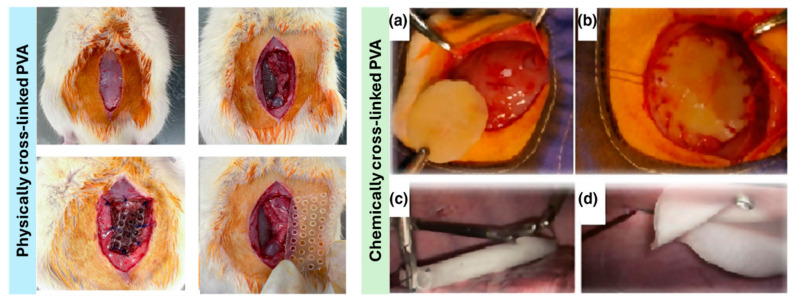
Chemically (GDA) cross-linked (**left**) and physically (freeze-thawed) (**right**) PVA hydrogel implantation in Wistar rats (**a**,**b**) and Swine (**c**,**d**) models. Note, surgery on swine models was performed laparoscopically. Adapted from Dorkhani et al. and Fehér et al. [35,91].

PVA meshes have been proved not only to be highly flexible, but to possess a mechanical strength satisfactory for hernia repair. They are also applicable in laparoscopic surgery, withstanding both the sutures and clips (Figure 9c,d). Furthermore, when compared to those of the polypropylene control, their anti-adhesion features are impressive. N-isopropylacrylamide-N-vinylpyrrolidone was successfully implemented via injection. Due to it being thermoresponsive, the gel solidified at body temperature. This breakthrough suggests that the need for traditional mesh implantation could be potentially eliminated. An injectable gel could be utilized, minimizing the operative times [88]. The reported advantageous features are summarized in Table 9.

Biological materials, being biodegradable, induce only mild inflammatory reactions, and often circumvent fibrous capsule formation. This would allow for true tissue integration without post-operative complications. These materials are highly popular for wound-healing applications. Unfortunately, as seen in Table 8, their tensile strength hinders their use for hernia treatment. The efforts to fabricate strong enough standalone hydrogels include gelatin [39], decellularized dermal matrices [92,93], and hyaluronic acid [63,93], collagen [98].

Interestingly, hyaluronic acid, which has been proven to provide tissue regeneration and wound healing, has also been investigated in combination with different cross-linking agents such as calcium alginate. Perhaps the most notably utilized material is resiline, a polymer obtained from insects and other arthropods, possessing mechanical properties similar to visceral organs and tissues [95].

Amongst all the biological membranes, resiline proved to be the strongest, with a tensile strength of 550 MPa, far exceeding those of gelatin, collagen, and even silk fibroin (Table 8). Overall, post-operative complications were not documented, with mild adhesion formations and minimal inflammatory reactions (Table 10). Furthermore, additional features, such as increased collagen I production, antibacterial effects, and low-level hemolysis, have also been reported (Table 8). However, apart from two studies, the in vivo experiments were rather short (<1 month); therefore, there is no significant evidence that hernia recurrence will be prevented.

## 7. Quo Vadis? Hydrogels vs. Surgical Meshes

Regarding tissue integration, hydrogels perform unequivocally better. By optimizing their physio-chemical properties [99], hydrogels can provide a favorable template for cell adhesion, proliferation, and differentiation for almost every single cell type in the human body [44]. In addition, they can also be designed to be anti-adhesive, preventing visceral adhesion and thus most of post-operative surgical mesh-associated complications can be avoided. At the same time, the lack of overwhelming inflammation (in contrast to surgical meshes) and granulation tissue formation make the need for a reliable mechanical performance more pronounced. Hydrogels have to support the abdominal wall until the innate tissues take over and closes the abdominal defect. While there have been developments to fabricate mechanically enhanced hydrogels [42,91,100], the meshes from extruded thermoplastic polymers are inevitably stronger. Thus, it seems more reasonable to perform studies assessing the minimal tensile strength required by a hydrogel to provide sufficient support and prevent hernia recurrence. Furthermore, regardless of the fabrication method, the mechanical performance should always be evaluated in a clinically relevant setting. Uniaxial tensile strength unfortunately does not depict the entire picture. More relevant examinations, such as ball bursts or suture retention, should be performed in the future (Figure 8).

Another interesting point is the fact that bilayer constructs have been extensively investigated, yet we could not find any significant transition to commercially available products. Perhaps the issue lies in the scaling up of fabrication. According to our findings, bilayer constructs are not providing the optimal, but rather a transient solution.

A rarely mentioned drawback of using hydrogel membranes is the risk of infection. Although some studies have shown antimicrobial properties in certain types of hydrogel, there is still a risk of bacterial colonization and infection [101]. Microbiological studies are not typically included in the works presented. On the other hand, surgical meshes have been associated with post-operative infection. In most cases, surgical site infection is not caused directly by the mesh. Typically, surgical site infection is caused by either endogenous factors (the patient’s skin flora), or from an opened visceral organ (typically the bowel). However, as these meshes can cause extreme inflammation and perforation close to the bowels, they could be considered as an indirect cause of infection. Nevertheless, adequate evidence was not found regarding specific risk factors (the surgical technique and the type of implanted mesh) regarding surgical site infection. While infection is an issue for surgical meshes, hydrogels could overcome this obstacle. Antibacterial hydrogels have been noticeably documented in recent years [101]. From antibiotics to antibacterial peptides and nanoparticles, hydrogels can be functionalized in a far simpler manner than their surgical mesh rivals.

Finally, to our dismay, we could not find any clinical trials examining standalone hydrogel membranes. Overall, the lack of comprehensive investigation supporting the clinically relevant mechanical performance is limiting their transition to clinical studies (Table 11).

## 8. Conclusions

Hydrogels are fascinating materials with numerous advantages. In the context of hernia repair, hydrogels can provide biocompatibility, regulate the immune response, and inhibit visceral adhesion formation, preventing post-operative complications. Their soft and flexibility nature not only provides a tissue-like template for cells, but can also make operative manipulation easier. Furthermore, compared to conventional surgical meshes their functionalization and drug incorporation potential are unparalleled. Advancements in cross-linking and hybrid hydrogel composites have improved strength, regulated biodegradation, and introduced antimicrobial and thermoresponsive properties. However, significant challenges persist, including poor mechanical properties, a lack of long-term in vivo experimental data, and susceptibility to bacterial colonization. Therefore, further research including application-specific mechanical characterization and long-term in vivo studies with large animal models are needed to fully validate the safety and effectiveness of hydrogel membranes before initiating any clinical trials.

## Figures and Tables

**Table 1:** Hernia mesh classification.

Hernia Mesh Classification			
By Origin	By Degradation	By Density (g/m^2^)	By Porosity (µm)
Synthetic Biological Composite	Non-Biodegradable Biodegradable Bioabsorbable (non-enzymatic degradation)	Heavyweight: >90 Medium Weight: 50–90 Light Weight: 35–50 Ultra-light weight: <35	Very Large Pore: >2000 Large Pore: 1000–2000 Medium Pore: 600–1000 Small Pore: 100–600 Microporous: <100

**Table 2:** Examples of cross-linking strategies [45,46].

Cross-Linking Method	Mechanism	Advantages	Disadvantages
Physical	Polymer chains are non-covalent Bonded via hydrogen bonding, ionic, hydrophobic interactions, or crystalline formation. Achieved by temperature or pH modification	No need for cross-linking agent Affordable	Bonds are weak and reversible resulting in a less stable membranes with poor mechanical performance compared to other methods
Cross-linker mediated	Covalent bonding between polymer chains with the help of cross-linking agents	Stable hydrogels with tuneable mechanical properties	Cross-linking agent may cause cytotoxicity and biocompatibility issues
UV mediated	Cross-linking is achieved using a photoinitiator and UV light	Spatial and temporal control over the cross-linking process, can be combined with 3D printing	Poor mechanical performance compared to chemical cross-linking Expensive
Enzyme mediated	Enzymes such as transglutaminase are exploited to form covalent bonds between polymers chains	High specificity and mild reaction conditions, suitable for biological systems.	Relatively slow reaction times and the cost of enzyme

**Table 3:** Experimental information of selected propylene–hydrogel bilayer systems (N/A: not available or not performed).

Polypropylene Mesh	Hydrogel Component	Cross-Linking Agent	Mechanical Studies	In Vivo Studies		Ref.
				Animal Model	Duration	
Gynecare Gynemes ps^®^ (Ethicon, J & J, Auneau, France)	Polysaccharide (pullulan-dextran 75:25)	Trisodium trimetaphosphate (STMP)	Compression strength (150–200 kN/m^2^)	Wistar Rats: subcutaneous pocket abdominal intramuscular placement	30 Days	[60]
Prolene^®^ (Ethicon, J & J, Somerville, NJ, USA)	N-vinylpyrrolidinone (NVP) N-butylmeth- acrylate (BMA) (1: 1)	Heat	N/A	Wistar Rats: Intra-abdominal placement	30 Days	[29]
Not specified	2-hydroxyethyl methacrylate (p(HEMA))	N/A	N/A	Dogs	20 days	[61]
Ultrapro^®^ mesh (Polypropylene-Poliglecaprone 25, Ethicon, Norderstedt, Germany)	PLGA/sP(EO-stat-PO) (Polylactide-co-glycolide/ Star-shaped poly(ethylene oxide-stat-propylene oxide))	Reactive isocyanate (NCO)	N/A	Chinchilla Bastard Rabbits	4 months	[62]
Optilene mesh elastic (B. Braun, Melsungen, Germany)	Poly(*N*-isopropylacrylamide) hyaluronan derivative (HApN)	N/A	N/A	New Zealand White rabbits: Mesh repair in preperitoneal position	14 days	[63]
Not specified	Silk cocoons of mulberry silkworm *Bombyx Mori*	N, N′-methylene bisacrylamide	N/A	Rabbits: Midline incision on the abdomen	1 month	[64]
Bard^®^ Mesh (BD, Franklin Lakes, NJ, USA)	Pectin-Honey based hydrogel	N/A	N/A	Wistar Rats: Midline incision on the abdomen	1 month	[65]
Not specified	Hyaluronic acid sodium (HA), Polyvinyl alcohol (PVA), 3,4-dihydroxyphenylacetic acid (DHPA)	Freeze thawing	Compression test (≈12–13 kPa)	Sprague-Dawley Rats: Midline incision on the abdomen	1 month	[66]
Not specified	Hydroxypropyl chitosan azide	UV light exposure	N/A	Sprague-Dawley Rats: Dorsal Subcutis and Leg Muscles implantation New Zealand White Rabbits: Intracutaneous Injections	180 days	[67]
Bard^®^ Mesh (BD, USA)	Oxidized-carboxymethylcellulose-*g*-dopamine (OCMC-DA) and Carboxymethyl chitosan (CMCS)	N/A	N/A	Piglets: Laparoscopy Implantation	1 year	[68]
Not specified	Synthetic, long chain polyol on a Polyurethane backbone	N/A	N/A	Sprague-Dawley rats: Dorsal Midline Incision	2 and 12 weeks	[69]
Not specified	Chondroitin Sulfate and Gelatin	Tannic acid	Cyclic tensile test (4.5–5 mN/cm)	Mice: Subcutaneous Implantation	21 days	[70]
Not specified	Pectin–Honey Based hydrogel	N/A	N/A	Sprague-Dawley Rats: Left Paramedian Skin Incision	14 days	[22]
Prolene^®^ (Non-absorbable Surgical Suture U.S.P., Healthium Medtech, Bengaluru, India)	Porcine Cholecystic Extracellular Matrix (CECM)	Formaldehyde	Uniaxial Tester (39.59 N)	Sprague-Dawley Rats Ventral Midline Skin Incision	16 weeks	[71]
Prolene^®^ (Ethicon, J & J, Somerville, MA, USA)	Sirolimus (SRL) hydrogel	N/A	N/A	Male BALB/c mice: Midline Laparotomy	24 weeks	[38]
Not specified	Aldehyde *Bletilla striata* polysaccharide (BSPA) modified chitosan (CS) hydrogel	Schiff Base Reaction	N/A	Sprague-Dawley Rats: Abdominal Wall Implantation	4 weeks	[72]
Flat hernia mesh (Condiner Medical, Changzhou, China)	Silk fibroid (*Bombyx mori*) and PLA	N/A	Bose Electroforce load testing (17–18 MPa)	Sprague-Dawley Rats: Abdominal Incision	90 days	[73]
Bard^®^ Mesh (BD, USA)	ECM from Pig Skin	N/A	Planar Biaxial Testing (≈85 KPa)	Rat: Ventral Midline Skin Incision	180 days	[74]
SIS Sterile Mesh (Beijing Biosis Healing Biological Technology Co., Ltd., Beijing, China)	Porcine Gelatin and Methacrylic anhydrite (GelMA)	Tannic acid (TA)	Tensile and adhesive test (≈3–4 MPa)	Sprague-Dawley Rats	14 days	[75]
Ultrapro^®^ mesh (Polypropylene-Poliglecaprone 25, Ethicon, Johnson & Johnson Medical, Wokingham, UK)	Collagen and Polyphosphate	N/A	MultiTest 2.5-xt Force (≈12–13 N/mm^2^)	N/A	N/A	[23]
Prolene^®^ Mesh (Ethicon—J & J)	Polyethylene Glycol (Coseal^®^)	N/A	N/A	New Zealand Albino Rabbits: Median Laparotomy Incision	30 days	[76]
Bard^®^ Mesh (BD, C.R. BARD-Davol, Providence, RI, USA)	ECM from Pig Skin	N/A	Biaxial tester (≈100 N/m)	Sprague-Dawley Rats: Ventral Midline Incision	35 days	[77]
High-weight- medium-sized pore mesh (Beijing TransEasy Medical Technology, Beijing, China)	Bacterial cellulose and Chitosan	N/A	N/A	Sprague-Dawley Rats: Abdominal Midline Skin incision	14 days	[78]
HERNI PRO, type P3 (Biosintex, Snagov, Romania)	(Methacryloyl gelatin/Methacryloylmucin) (GelMA/MuMA)	EDC/NHS cross-linking system	Cyclic traction (BioDynamic 5210)	N/A	N/A	[79]
Large pore mesh (Changzhou Runyuan Medical Supplies Technology, Changzhou, China)	Alginate	N/A	Tensile Test (≈50 KPa)	New Zealand White Rabbits: Abdominal Skin Midline Incision	30 days	[80]
Bard^®^ Mesh (BD, C. R. Bard, Inc., Franklin Lakes, NJ, USA)	Poly[poly(ethylene glycol) methacrylate-co-dopamine methacrylamide] (PEDMA)	N/A	N/A	Sprague-Dawley Rats: Midline Incision	30 days	[81]

**Table 4:** Advantageous features of hydrogel component utilized in polypropylene bilayer constructs.

Hydrogel Component	Features	Reference
Polysaccharide (pullulan-dextran 75:25)	Cytocompatibility Biocompatibility Decreased inflammation Decreased Foreign Body Reaction	[60]
N-vinylpyrrolidinone (NVP) N-butylmeth- acrylate (BMA) (1: 1)	Initial acute inflammation Mild inflammation after 30 days Decreased adhesion formation Fibrous capsule formation	[29]
2-hydroxyethyl methacrylate (p(HEMA))	Decreased visceral adhesion formation	[61]
PLGA/sP (EO-stat-PO)	Enhanced biocompatibility	[62]
Poly(*N*-isopropylacrylamide) hyaluronan derivative (HApN)	Drug carrier system Infection prevention (Rifampicin) Enhanced tissue integration	[63]
Silk cocoons of mulberry silkworm *Bombyx Mori*	Supported cell growth Decreased adhesion formation Minimum fibrotic changes Decreased inflammation	[64]
Pectin–Honey Hydrogels	Improved peritoneal regeneration Improved tissue healing and integration	[65]
Hyaluronic acid sodium (HA) Polyvinyl alcohol (PVA) 3,4-dihydroxyphenylacetic acid (DHPA)	Excellent coating stability Hemocompatibility Non-cytotoxicity Reduced inflammation and adhesion formation	[66]
Hydroxypropyl chitosan azide	Biocompatibility Good adherence to PPM Promote wound healing Reduced adhesion formation Fibrin lysozyme activity Potential antibacterial effect	[67]
Oxidized-carboxymethylcellulose-*g*-dopamine (OCMC-DA) and Carboxymethyl chitosan (CMCS)	Excellent biocompatibility Reduced adhesion formation Decreased inflammation Decreased collagen deposition	[68]
Synthetic, long chain polyol on a Polyurethane backbone	Decreased foreign body giant cells Decreased fibrosis Decreased oxidative damage, fibroblast accumulation, apoptosis, and macrophages	[69]
Chondroitin Sulfate and Gelatin	Reduced inflammation Reduced collagen deposition Increased vascularization and tissue regeneration	[70]
Pectin–Honey Based hydrogel	Decreased adhesion formation	[22]
Porcine Cholecystic Extracellular Matrix (CECM)	Biocompatibility Decreased inflammation and cytotoxicity Deposition of Collagen type I	[71]
Sirolimus (SRL) hydrogel	Decreased adhesion formation	[38]
Aldehyde *Bletilla striata* polysaccharide (BSPA) modified chitosan (CS) hydrogel	Biocompatibility Anti-adhesion properties Decreased inflammation Enhanced collagen deposition Enhanced neovascularization	[72]
Silk fibroid from silkworm (*Bombyx mori*) cocoons and PLA	High mechanical strength Reduced inflammation Inhibition of adhesion formation Promotion of fibroblast proliferation	[73]
ECM from Pig Skin	Decreased inflammation Decreased collagen deposition No fibrous capsule formation	[74]
Porcine Gelatin and Methacrylic anhydrite (GelMA)	Excellent wet adhesiveness Antioxidant, and antibacterial ability Excellent biodegradability, biocompatibility, and immunoregulatory activity	[75]
Collagen and PolyPhosphate	Biocompatibility Good mechanical stability Improved cell attachment and growth	[23]
Polyethylene Glycol (Coseal^®^)	Decreased adhesion formation	[76]
ECM from Pig Skin	Decreased inflammatory response and cell accumulation Fewer foreign body giant cells Loose connective tissue replacement Good mechanical stability	[77]
Bacterial cellulose and Chitosan	Biocompatibility Decreased adhesion formation Decreased inflammatory response Better tissue ingrowth	[78]
GelMA (methacryloyl gelatin)/MuMA (methacryloyl mucin)	Modulated PP meshes integration Enhanced cell interactivity Enhanced wound healing	[79]
Alginate	Excellent structural stability and biocompatibility Prevention of visceral adhesions Promote mesh integration	[80]
Copolymer: Poly[poly(ethylene glycol) methacrylate-co-dopamine methacrylamide] (PEDMA)	Biocompatibility Decreased adhesion formation Reduced inflammation	[81]

**Table 5:** Experimental information of selected PE, PTFE, and PU bilayer constructs (N/A: not available or not performed).

Main Component	Hydrogel Component	Cross-Linking Agent	Mechanical Studies	In Vivo Studies		Ref.
				Model	Duration	
Polyester mesh (specifics not available)	Blend solution of poly (vinyl alcohol) and poly (vinyl pyrrolidone) (PVA/PVP)	Freeze-thawing	Materials testing machine (QTest; MTS Corp., Minneapolis, MN, USA) (≈1.3 N)	Sprague-Dawley Rats: Midline Ventral Laparotomy	7 days	[37]
Polyurethane electrospun fibers	Methacrylinated Gelatin and Nanosilver coated	N/A	N/A	Sprague-Dawley rats: Lower Abdominal Wall Insertion	4 weeks	[82]
Polyester mesh MotifMesh^®^ (Proxy Biomedical)	Gelatin (bovine bone)	Glutaraldehyde	N/A	Mice: Subcutaneous pocket	21 days	[86]
e-PTFE mesh Bard composix kugel hernia (Mesh Davol Inc., Warwick, RI, USA)	Poloxamine hydrogel: PPO-PEO (propylene oxide—polyethylene oxide)	Dithiothreitol	Uniaxial lap shear testing (≈70 KPa)	N/A	N/A	[87]

**Table 6:** Advantageous features hydrogel component utilized in PE, PU, and PTFE bilayer co structs.

Hydrogel Component	Features	Reference
Poly (vinyl alcohol) and poly (vinyl pyrrolidone) (PVA/PVP)	Milder Cellular Adhesions	[37]
Methacrylinated Gelatin and Nano silver	Reduced adhesions Provide necessary mechanical support Good antimicrobial activity Graft accommodation	[82]
Gelatin (bovine bone)	Increased angiogenesis	[86]
Poloxamine hydrogel: PPO-PEO (propylene oxide—polyethylene oxide)	Improved adherence between the mesh and hydrogel	[87]

**Table 9:** Advantageous features of selected of selected standalone synthetic hydrogel membranes.

Polymer	Features	Reference
Poly(vinyl alcohol)	Biocompatibility Good integration No foreign body reaction Non-adhesive and non-toxic	[35]
	Increased mechanical strength Withstand sutures	[42]
	Does not favor cell adhesion Good integration Reduced inflammation and adhesion	[89]
	Reduced adhesion Improved fibroblast adhesions and tissue growth Anti-deformation and high mechanical strength	[90]
	Good water update capacity and wetting behavior High mechanical strength No cytotoxicity Reduced adhesion and granuloma formation Greater tissue integration Higher neovascularization Enhanced collagen deposition	[91]
Poly (NIPAAM-co-VP-co-MAPLA)	Constructive foreign body reaction Reduced inflammation and fibrosis Promote cell infiltration	[88]

**Table 10:** Advantageous features of selected standalone biological hydrogel membranes.

Polymer	Features	Reference
Poly (g-glutamic acid) and Gelatin	High strength, super-toughness No visceral adhesion formation	[85]
Gelatin and Acid Anhydride (GelMA)	Good cohesion and adaptation capability Decreased visceral adhesion	[39]
Decellularized ECM	Cytocompatibility Biocompatibility Decreased inflammation Reduced Foreign Body Reaction	[92]
Tyramine-substituted hyaluronan (THA)	Biocompatibility Mimics the ECM Good mechanical stability	[93]
Hyaluronic acid (HA) derivative	Enhanced degradation at lower cross-linking density	[41]
	Higher modulus and stability at 37 °C Low hemolysis Reduced adhesion formation	[94]
Resilin	Reduced inflammatory response Upregulate collage type 1/3 Promote fascia regeneration	[95]
Silk fibroin	Inhibits targeted microorganism Expression of collagen type I Fibroblast cell migration Accelerate wound closure	[96]
Bovine collagen	Improved mechanical strength Antibacterial activity Non-toxicity and cytocompatibility Promote cell proliferation and wound healing	[97]

**Table 11:** Advantages and disadvantages of hydrogels and meshes.

Hydrogels	Surgical Meshes
Tissue-like consistency Biodegradability with true tissue integration Functionalization and drug delivery possibilities	Withstand surgical manipulation Superior mechanical performance Low incidence of hernia recurrence Simple sterilization
Poor mechanical performance Lack of long-term in vivo data	Typically composed of non-degradable material Known for adhesion formation, infection and other post-operative complications Difficult to functionalize

## Data Availability

No new data were created or analyzed in this study. Data sharing is not applicable to this article.
